# Demographic Variation in the Frequency of Gliomas in Florida

**DOI:** 10.3390/medicina55010005

**Published:** 2019-01-04

**Authors:** Dharam Persaud-Sharma, Joseph Burns, Jeran Trangle, Grettel Castro, Noel Barengo, Sabyasachi Moulik, Juan Manuel Lozano

**Affiliations:** Herbert Wertheim College of Medicine Miami, Florida International University, Miami, FL 33199, USA; jburn052@med.fiu.edu (J.B.); jtran009@med.fiu.edu (J.T.); gcastro@fiu.edu (G.C.); nbarengo@fiu.edu (N.B.); smoulik@fiu.edu (S.M.); jlozanol@fiu.edu (J.M.L.)

**Keywords:** glioma, brain cancer, race, populations, Florida, demographics

## Abstract

*Background and objectives:* Glial brain cancers affect nearly 20,000 individuals in the United States (USA) annually. SEER database data exploring the relationship between race and gliomas is now available and have shown that cerebral gliomas occur at a higher frequency in Caucasian men. However, such analyses did not include demographic data specific to the state of Florida. This study assessed the association between race and glial vs. non-glial Central Nervous System (CNS) cancers in Florida, USA. *Materials and Methods:* This case-control study utilized the Florida Cancer Data Registry (FCDS), in which race was considered the exposure and development of glioma as the measured outcome. The sample was comprised of patients in Florida diagnosed with brain tumors from 1981 to 2013. Relative racial frequencies were compared between patients with glial brain tumors and those with other CNS tumors. Data was analyzed using logistic regression in order to determine any associations between race and frequency of diagnosis adjusting for several confounders (age, sex, smoking status, year of diagnosis, and insurance status). *Results:* Between 1981 and 2013 a total of 14,092 patients meeting the inclusion and exclusion criteria were diagnosed in Florida with a primary brain tumor. Being of non-white race was associated with 60% decreased odds of glioma diagnosis compared to the reference white population (adjusted OR 0.4, 95% CI 0.34–0.47). Secondary findings include associations between increasing age and male sex with increased odds of glioma diagnosis. Decreased adjusted odds of glioma diagnosis were found with former smoking status (reference non-smokers), diagnosis between 2001 and 2010 (reference 1981–1990), and Medicaid or Medicare insurance (reference private insurance). Hispanic ethnicity, current smoking status, no insurance/self-pay, and geographical location (urban vs. rural) all had no association with glioma diagnosis. *Conclusions:* These findings are consistent with and help reinforce previous studies utilizing national databases (SEER) which also showed increasing odds of glioma diagnosis in older white males. Various potential explanations for these findings include genetic predisposition, lifestyle and behavioral factors, and socioeconomic status, including access to healthcare. Future research aims at identifying potential genetic etiologies.

## 1. Introduction

Cancers of the brain affect more than 20,000 American families annually [1]. Among those affected, nearly 10,000 cases are gliomas [2]. Cancers of glial origin, or gliomas, arise from glia, the supporting cells of the brain. These cells give rise to more than 80% of all primary brain malignancies [3]. Among the most common types of brain cancers, these tumors may be further classified as astrocytomas, arising from astrocytes, oligodendrogliomas, from oligodendrocytes, and ependymomas from ependymal cells [4]. There are several hypotheses proposing models for the development of gliomas [5]. The most commonly accepted of these echoes the Knudson two-hit hypothesis [6]. This theory proposes that genetic changes, whether inherited or environmental, alter critical genes affecting the regulatory capacity of cells. When both copies of such genes are affected, unchecked proliferation of glial cells gives way to the formation of gliomas. Though this pathway has not been proven in gliomas, several critical genetic mutations have been identified [7].

In 2017, the classification system for the diagnosis of gliomas was revised to include these biochemical changes in addition to cell type, location, and grade [4,8]. Among many changes are the addition of isocitrate dehydrogenase (IDH) mutation status, 1p/19q deletion status, ATP-dependent helicase (ATRX) function, p53 mutation, and nitric oxide synthase (NOS) status [9]. As the understanding of the role of these mutations in the pathogenesis of glioma increases, and as we prepare ourselves to further direct treatment based on genetic and epigenetic changes, it is more important than ever that scientists and clinicians aim for timely diagnosis and treatment of glial cancers [9].

Despite advances in the diagnosis and treatment of these cancers, they are notoriously aggressive, invasive, and destructive [10]. Of the 10,000 cases of malignant gliomas diagnosed in the United States, only 25% survive one year after their diagnosis [3]. The mean survival time of only 9 to 12 months has remained stable despite intensity of treatment. The high morbidity and mortality associated with the diagnosis of glioma is largely due to the lack of a uniform approach to treatment [5]. Treatment is often complicated by molecular phenotypic variation necessitating a multi-modal treatment approach [11]. This is further complicated by the heterogeneous anatomy of these tumors. Often, advanced tumors may lie in proximity to the hypothalamus, requiring an exquisitely delicate surgical approach [12]. Comprehensive therapy often entails surgery, radiotherapy, and chemotherapy [7].

The overall incidence of cancers of the brain has increased over the last thirty years but has recently begun a gradual decline [3]. Despite this statistic, the incidence of glioblastoma is increasing [3]. National data on the diagnosis of glial cancers is maintained by the National Cancer Data System. This data generated a robust analysis of demographic associations with frequency of diagnosis of glioma relative to non-glial brain cancers from 1997 to 2007. This study demonstrated that the frequency of gliomas is highest in white Americans, with lower rates in blacks, and the least frequent in East and Southeast Asians [1]. Further, this analysis revealed that men were more frequently diagnosed than women, and that elderly individuals were more likely to be diagnosed than younger adults [1]. Those residing in more metropolitan communities were more likely to be diagnosed than those in rural areas [1]. 

It is proposed that these associations may not necessarily reflect a purely biological attribution [5]. With increased use of computerized tomography (CT), the incidence of cancers of the brain skyrocketed in the 1970s [3]. Further, the demographic most likely to have access to CT scans likely correlates well with the data [5]. This is to say that older white men in metropolitan areas are more likely than other subgroups to be diagnosed with glial cancers and yet may not necessarily represent a difference in biological association, but rather missed opportunities for diagnosis in other populations [5].

The aim of our study is to investigate the association between race and the type of brain cancer (gliomas vs. non-glial brain cancers) in the state of Florida from 1981 to 2016. In achieving this objective, it is the larger goal of the authors to generate meaningful data to support clinical screening and treatment [12,13]. 

## 2. Materials and Methods

For this study, we conducted a case–control study utilizing publicly available data from the Florida Cancer Dataset [14]. In the database, glioma tumor data was available using the ICD codes C710–719 from their earliest recording through 31 December 2016.

The population of interest was patients diagnosed with brain tumors in the state of Florida. This population was divided into patients who developed “gliomas”, and our comparator group, those who developed “non-glioma CNS tumors”. This classification was based on the following ICD-03 codes: Gliomas: C710–C19 with morphology codes 9380, 9400, 9410, 9411, 9420, 9450, 9460, 9451; Non-Gliomas: 9440, 9441, 9442. The control group consisted of all patients from Florida with non-glial primary brain tumor diagnoses available in FCDS. They were unmatched, as the potential confounders did not allow proper cross-matching with the glioma group. Exclusion criteria included duplicate records, data from patients less than 18 years old at diagnosis, and records with a missing or “unknown” race value.

The dependent variable in this study is the type of brain tumor, classified in two groups: patients with glioma brain tumors and patients with non-glioma brain tumors. The independent variable is the racial status, which was aggregated as white or non-white. The FCDS database utilized the 2000 Census and Bureau of Vital Statistics to encode the race variable with respondents self-reporting their race. The FCDS database fields NAACCR 160, 161, 162, 163, 164 correspond to the following racial groups: white, black, other, and unknown. Thus “white” consisted of the “white” group and non-white included “black” and “other”, with all data records with a value of “unknown” excluded per criteria. Potential confounders included age, gender, Hispanic ethnicity, smoking status, year of diagnosis, insurance status, and geographic region (urban or rural).

Data were analyzed using STATA version 14. Measurements of central tendency and dispersion were reported for continuous variables. Absolute and relative frequencies were reported for categorical variables. Each instance of a cancer diagnosis was characterized by the patient’s racial category. For both glial and non-glial cancers, the frequency of glioma tumors for each racial group, as well as the frequency of non-glioma CNS tumors were calculated. We further subdivided our cases and control groups to analyze relative frequency of diagnosis between races based upon age, gender, Hispanic ethnicity, smoking status, year of diagnosis, insurance status, and geographic region, defined by the Rural Health Research Center [7]. Chi squared test (for categorical variables) and Student’s *t*-test (for continuous variables) were used to assess associations between variables of interest and race. A logistic regression model was utilized to compute unadjusted and adjusted odd ratios (OR) and corresponding 95% confidence intervals (CI). Variables found to be associated with the independent and dependent variables were deemed potential confounders and included in the adjusted model. An alpha level of 0.05 or lower was used to assess statistical significance. 

The FCDS database is a publicly available, de-identified database maintained by the Florida Department of Health in conjunction with the University of Miami Sylvester Comprehensive Cancer Center. Access and use of FCDS data were obtained via agreement with the Florida International University Herbert Wertheim College of Medicine and signed certificates of confidentiality. As the data was de-identified and retrospective, this study was classified as non-human subject research and thus exempt from requiring IRB approval.

## 3. Results

**Figure 1:**
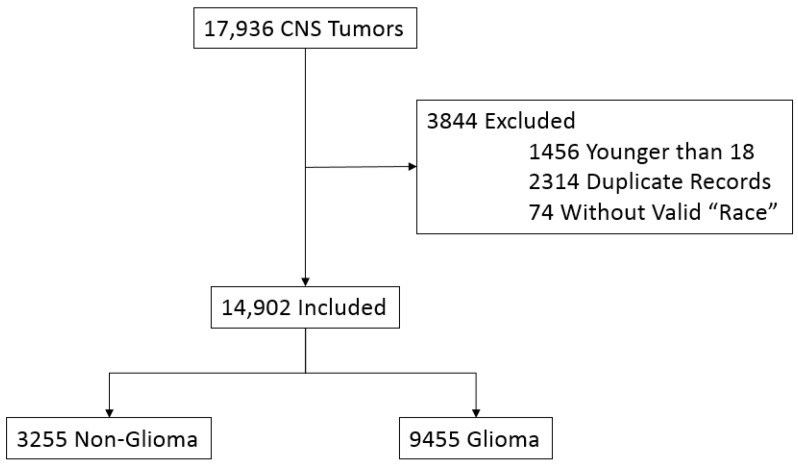
Development of sample population of Floridians diagnosed with gliomas and non-glial primary CNS tumors from 1981 to 2013 based on inclusion and exclusion criteria.

Figure 1 shows the process for selecting the patients included in the study. Between 1982 and 2013 a total of 17,936 patients were diagnosed in Florida with a primary brain tumor. After applying the selection criteria, 14,092 patients were retained for the study. Information regarding patients’ characteristics is summarized in Table 1. Mean age at diagnosis of a glioma was older in white patients (55.9 years) as compared to non-whites (48.5 years) (*p* ≤ 0.001). There were also significant differences between races in the distributions of smoking status, Hispanic ethnicity, year of diagnosis, insurance status, and geographical region (*p* ≤ 0.001). On the contrary, there was no significant difference with respect to sex between white and non-white individuals (*p* = 0.128).

As detailed in Table 2, there was a significant difference in racial frequency according to the type of brain tumor (glioma vs non-glioma) (*p* ≤ 0.001). Additionally, the two groups also showed statistically significant differences according to age, sex, smoking status, year of diagnosis, and insurance status (*p* ≤ 0.001). There were no statistically significant differences by geographical region (*p* = 0.196) or Hispanic ethnicity (*p* = 0.996).

Table 3 shows the unadjusted and adjusted associations between the different demographic characteristics and the diagnosis of glioma versus non-glial CNS tumor. Several of the independent variables studied were significantly associated with the type of CNS tumor. The adjusted odds of having a glioma is 60% less in non-whites compared with whites (55% less using unadjusted model). After adjustment for the covariates the odds of being diagnosed with glioma decreases by 3% with each year of advancing age (95% CI 0.97–0.98). Further, the adjusted odds of having a glioma is 27% less in females compared to males. The adjusted odds of glioma diagnosis is the same in current smokers as compared with those who have never smoked; however, former smokers had a 14% decreased odds of being diagnosed with glioma. Additionally, the odds of glioma diagnosis from 2001–2010 decreased by 68% (95% CI 0.14–0.72) relative to 1981–1990. Odds of glioma diagnoses were similar between 1991–2000 and 2011–2013 relative to the reference of the 1981–1990 decade. Those insured by Medicare and Medicaid were 20% (95% CI 0.70–0.93) and 29% (95% CI 0.58–0.86) less likely to be diagnosed with glioma than those with private insurance, respectively. After adjusting to exclude insurance status, the only change in significance occurred in the year of diagnosis, altering the OR to 0.47 (95% CI 0.40–0.55) from 1991–2000 and 0.39 (0.32–0.48) between 2011 and 2013. There were no significant associations between Hispanic ethnicity, current smoking status, and the geographic region with tumor type.

## 4. Discussion

Our investigation regarding the relationship between diagnosis of the type of CNS tumors and race in Florida uncovered a significant increase in glioma diagnosis in the white population compared with the non-white population. This effect was demonstrated even after controlling for the potential confounders of age, sex, smoking status, year of diagnosis, and insurance status. Other factors such as age, sex, and insurance status were also independently associated with significant difference in glioma cancer rates; however, none had as large an effect as race. 

Our findings are consistent with previously published data by Ostrom et al. and Dubrow et al. [1,3], which both demonstrated increased odds of glioma diagnosis in whites when compared to non-whites. This is to say, the demonstration of older white men as the demographic most frequently diagnosed with glial cancers in Florida is consistent with current literature. Unfortunately, given the paucity of studies with comprehensive data sources, we are unable to place some of the secondary findings between insurance status and glioma, and year of diagnosis and glioma into the broader context of scientific literature.

There are numerous potential underlying explanations for the racial effect this study uncovered. It is hypothesized that these differences may be attributable to underlying genetic differences or to the social determinants of health [13]. Multiple genes have been associated with the development of gliomas and further research may demonstrate a propensity of these mutations in white Americans compared to non-white Americans [4]. Regarding the latter, it is well substantiated that non-white Americans are frequently under-diagnosed due to lack of access to health services [13]. 

Another possible rationale for this finding may include socioeconomic status and lack of access to healthcare, an endemic public health challenge affecting non-white communities across the United States. Additionally, the state of Florida is predominantly categorized as “white” by the US Census, with 2017 estimates that 77% of the population identifies as “white” [13]. This fact, compounded with racial differences in access, could contribute to the racial effect. This health inequity is perpetuated by several underlying factors, including historical trauma resulting in avoidance of medical professionals, cost barriers, and generalized impaired access to care [15,16]. These factors could cause those of low socioeconomic status, who tend disproportionately to be racial minorities, to be underdiagnosed and undercounted in the FCDS database. Further support for the impact of socioeconomic status is garnered from the analysis of insurance status. The analysis of collected data supports this by showing that those with private insurance are more likely to be diagnosed with a glioma compared to those covered by Medicaid and Medicare. Explanations for the delayed age of diagnosis are non-specific, though the molecular development of cancers center on the accumulation of genetic mutations that necessitates an element of time and could play a role. The underlying causes of glioma are poorly understood, including the observed relationship with cigarette smoking. Though several associated genes have been identified, including survival and proliferation factors, investigation in this field may generate valuable information in the identification and treatment of those affected by gliomas [16]. It is hypothesized that analysis of other behavioral and genetic features may reveal valuable clinical and biological information in the identification and treatment of glial cancers. Further studies should attempt to link genetic factors with glioma development and diagnosis. Numerous large-scale cohort studies with whole-genome sequencing data are being developed for various investigations and could prove an invaluable tool in uncovering genetic markers associated with glioma development.

Secondary findings from this investigation include significant associations between increased age, male gender, former smoking status, diagnosis from 2001 to 2010, and being insured by Medicare or Medicaid with higher rates of glioma. The significant findings between age and glioma status echo similar results among many other types of cancer, where greater age allows an increased number of genetic mutations that may ultimately lead to cancer. Several different lifestyle choices may contribute to the development of gliomas, including smoking habits. It is somewhat counterintuitive that former smoking status significantly decreased the odds of glioma; however, given the 95% CI interval upper range is 0.99, it is not outside the realm of possibility that this finding is explained by random or sampling variation. The fact that no relationship was found with current smoking status further diminishes the strength of this association. Additionally, smoking status is traditionally associated with increased and not decreased rates of cancer. This finding is disputed by two cohort studies published by Holick et al., 2007, which concluded that there is strong evidence that cigarette smoking is not associated with an increased risk of adult gliomas [17]. A more recent survey of literature by Shao et al., 2016, further supported this conclusion by an investigative method of a dose-response meta-analysis of case-control and cohort studies of cigarette smoking and glioma [18].

From the general survey of available data, neither the geographical region of a patient’s residence, nor being of Hispanic ethnicity were associated with any specific type of CNS tumor. This finding was of particular interest since our hypothesis investigated race as an independent variable, and both geographical region and Hispanic ethnicity have similar relationships to race. For example, many regions are racially dominated by primarily one race with relatively few areas of heterogeneous populations. The lack of these associations generated a corollary exploration to investigate the relationship of geographical location in Florida with glioma diagnosis. In doing so, all zip codes in Florida were divided into regions of population density based upon Version 2.0 of the Rural-Urban Commuting Areas (RUCA) developed in conjunction with the University of Washington and the US Office of Rural Health Policy (ORHP) [19]. This is an interesting note because the criteria for a rural area classification included isolated, small rural, and large rural areas. Thus, while one can inquire about possible environmental exposures or hazards that could potentially trigger genetic alterations leading to glioma development by mechanisms similar to the Knudson-two hit hypothesis or by means of primary development, this investigation was unable to definitively answer these questions [5]. 

Naturally, this study has some limitations. The FCDS dataset utilized for this investigation did not have data regarding alcohol use or socioeconomic status, limiting the model’s ability to account for these potential confounders. While the study had a large population that met both the selection criteria, various subgroup analyses were not possible given the small numbers of participants with those characteristics. For example, while there was more data regarding race available, the small numbers of non-whites (Hmong, Thai, Laotian, Pakistani, etc.) necessitated grouping. On the other hand, the large number of glioma cancers found in the FCDS data set, despite their relative rarity, provide robust power and offer compelling evidence of a real effect. Lastly, the study was limited by the quality of the FCDS with inter-observer pathology variability likely having a minimal, but unmeasurable impact.

## 5. Conclusions

This analysis serves to reflect the work of the surveillance and reporting efforts for institutions throughout the state of Florida. Our study effectively describes the racial demographic most likely to be diagnosed with having gliomas in Florida, which is data that was previously unpublished. Beyond demography, we are able to construct a complete picture of the individuals most frequently diagnosed based on the analysis of multiple factors, including geography, smoking status, insurance status, gender, and age. By understanding this data, we may more effectively target individuals for screening. Further, this analysis assists in the identification of populations that may be underdiagnosed with tumors of glial origin.

## Figures and Tables

**Table 1:** Characteristics of the study participants by race in USA from 1981 to 2013.

Characteristics	Race		*p*-Value
	Caucasian	African-American	
	N (%)	N (%)	
Age ^1^ (Years)—Mean (SD ^2^)	55.9 (18.2)	48.5 (16.8)	≤0.001
Gender			0.128
Male	6793 (53.5)	715 (51.4)	
Female	5902 (46.5)	677 (48.6)	
Hispanic Ethnicity			
Hispanic	1777 (14.1)	68 (5.0)	
Non-Hispanic	10791 (85.9)	1306 (95.1)	
Smoking Status			≤0.001
Never Smoker	5716 (58.4)	726 (68.2)	
Current Smoker	1966 (20.1)	199 (18.7)	
Former Smoker	2113 (21.6)	140 (13.2)	
Year of Diagnosis			≤0.001
1981–1990	3037 (23.9)	175 (12.6)	
1991–2000	3788 (29.8)	383 (27.5)	
2001–2010	4771 (37.6)	672 (48.3)	
2011–2013	1104 (8.7)	162 (11.6)	
Insurance Status			≤0.001
Not Insured/Self Pay	575 (7.3)	755 (14.8	
Medicare	2803 (35.6)	255 (24.4)	
Medicaid	613 (7.8)	183 (17.5)	
Other Public Insurance	193 (2.5)	22 (2.1)	
Private Insurance	3684 (42.8)	431 (41.2)	
Region			≤0.001
Urban	11,450 (94.3)	1312 (96.0)	
Rural	692 (5.7)	55 (4)	

^1^ Age analysis using *t*-test, all other variables Chi Squared analysis; ^2^ Standard deviation.

**Table 2:** Population characteristics according to diagnosis of glioma versus non-glial CNS tumors in USA from 1981 to 2013.

Characteristics	Type of CNS ^1^ Tumor		*p*-Value
	Non-Glioma	Glioma	
	N (%)	N (%)	
**Race**			≤0.001
Caucasian	3255 (25.6)	9445 (74.4)	
African-American	606 (43.5)	786 (56.5)	
**Age ^2^ (Years)—Mean (SD ^3^)**	59.6 (17.7)	53.5 (18.1)	≤0.001
**Gender**			≤0.001
Male	1810 (24.1)	5698 (75.9)	
Female	2047 (31.1)	4532 (68.9)	
**Hispanic Ethnicity**			0.886
Hispanic	509 (27.6)	1336 (72.4)	
Non-Hispanic	3318 (27.4)	8779 (72.6)	
**Smoking Status**			≤0.001
Never Smoker	1739 (27.0)	4703 (73.0)	
Current Smoker	515 (23.8)	1650 (76.2)	
Former Smoker	684 (30.4)	1569 (69.6)	
**Year of Diagnosis**			≤0.001
1981–1990	397 (12.4)	2815 (87.6)	
1991–2000	951 (22.8)	3220 (77.2)	
2001–2010	2172 (39.9)	3271 (60.1)	
2011–2013	341 (26.9)	925 (73.1)	
**Insurance Status**			≤0.001
Not Insured/Self Pay	199 (27.3)	531 (72.7)	
Medicare	1349 (44.1)	179 (55.9)	
Medicaid	268 (33.7)	528 (66.3)	
Other Public Insurance	56 (26.1)	159 (74.0)	
Private Insurance	1115 (27.1)	3000 (72.9)	
**Region**			0.196
Urban	3585 (28.1)	9177 (71.9)	
Rural	204 (27.3)	542 (72.7)	

^1^ Central Nervous System; ^2^ Age analysis using *T*-test, all other variables Chi Squared analysis; ^3^ Standard Deviation.

**Table 3:** Unadjusted and adjusted associations between population characteristics and odds of diagnosis of glioma and non-glial CNS tumors.

Characteristics	Unadjusted	Adjusted	*p*-Value	Adjusted without Insurance	*p*-Value
	OR ^1^ (95% CI ^2^)	OR (95% CI)		OR (95% CI)	
Race					
Caucasian	Reference	Reference		Reference	
African American	0.45 (0.4–0.5)	0.40 (0.34–0.47)	≤0.001	0.42 (0.36–0.48)	≤0.001
Age	0.98 (0.98–0.98)	0.97 (0.97–0.98)	≤0.001	0.98 (0.97–0.98)	≤0.001
Sex					
Male	Reference	Reference		Reference	
Female	0.70 (0.65–0.76)	0.73 (0.65–0.81)	≤0.001	0.81 (0.74–0.90)	≤0.001
Hispanic					
Non-Hispanic	Reference	Reference		Reference	
Hispanic	0.99 (0.89–1.10)	0.90 (0.77–1.06)	0.197	0.81 (0.71–0.92)	0.002
Smoking Status					
Never Smoker	Reference	Reference		Reference	
Current Smoker	1.18 (1.06–1.33)	1.00 (0.86–1.16)	0.956	0.93 (0.82–1.05)	0.235
Former Smoker	0.85 (0.76–0.94)	0.86 (0.76–0.99)	0.031	0.88 (0.79–0.99)	0.034
Year of Diagnosis					
1981–1990	Reference	Reference		Reference	
1991–2000	0.48 (0.42–0.54)	0.67 (0.30–1.53)	0.346	0.47 (0.40–0.55)	≤0.001
2001–2010	0.21 (0.19–0.24)	0.32 (0.14–0.72)	0.006	0.22 (0.19–0.26)	≤0.001
2011–2013	0.38 (0.33–0.45)	0.57 (0.25–1.29)	0.176	0.39 (0.32–0.48)	≤0.001
Insurance Status					
Not Insured/Self Pay	0.99 (0.83–1.18)	0.92 (0.74–1.14)	0.460	-	
Medicare	0.47 (0.43–0.52)	0.80 (0.70–0.93)	0.004	-	
Medicaid	0.73 (0.62–0.86)	0.71 (0.58–0.86)	0.001	-	
Other Public Insurance	1.06 (0.77–1.44)	1.14 (0.78–1.67)	0.505	-	
Private Insurance	Reference	Reference		Reference	
Region					
Urban	Reference	Reference		Reference	
Rural	1.04 (0.88–1.23)	0.96 (0.77–1.20)	0.713	1.02 (0.84–1.25)	0.821

^1^ Odds Ratio; ^2^ Confidence Interval.

## References

[1] Ostrom Q.T., Gittleman H., Fulop J., Lio M., Blanda R., Kromer C., Wolinsky Y., Kruchko C., Barnholts-Sloan J.S. (2015). CBTRUS Statistical Report: Primary Brain and Central Nervous System Tumors Diagnosed in the United States in 2008–2012. Neuro Oncol..

[2] Stein R. (2008). Malignant Gliomas Affect About 10,000 Americans Annually. The Washington Post.

[3] Dubrow R., Darefsky A. (2011). Demographic variation in incidence of adult glioma by subtype, United States, 1992–2007. BMC Cancer.

[4] Louis D.N., Perry A., Reifenberger G., von Deimling A., Figarella-Branger D., Cavenee W.K., Ohqaki H., Wiestler O.T., Kleihues P., Ellison D.W. (2016). The 2016 World Health Organization Classification of Tumor of the Central Nervous System: A Summary. Acta Neuropathol..

[5] Persaud-Sharma D., Burns J., Trangle J., Moulik S. (2017). Disparities in Brain Cancer in the United States: A Literature Review of Gliomas. Med. Sci..

[6] Knudson A.G. (1971). Mutation and cancer: Statistical study of retinoblastoma. Proc. Natl. Acad. Sci. USA.

[7] Chang H.J., Burke A.E., Glass R.M. (2010). Gliomas. JAMA.

[8] Louis D.N., Ohgaki H., Wiestler O.D., Cavenee W.K., World Health Organization (2016). WHO Classification of Tumors of the Central Nervous System.

[9] Komori T. (2017). The 2016 WHO Classification of Tumors of the Central Nervous System: The Major Points of Revision. Neurol. Med. Chir..

[10] Maher E. (2001). Malignant glioma: Genetics and biology of a grave matter. Genes Dev..

[11] Ducray F., Idbaih A., Wang X., Cheneau C., Labussiere M., Sanson M. (2011). Predictive and prognostic factors for gliomas. Expert Rev. Anticancer Ther..

[12] Ampie L., Choy W., Lamano J.B., Kesavabhotla K., Mao Q., Parsa A.T., Bloch O. (2015). Prognostic factors for recurrence and complications in the surgical management of primary chordoid gliomas: A systematic review of literature. Clin. Neurol. Neursurg..

[13] US Census Bureau Quickfacts Florida. https://www.census.gov/quickfacts/fl#qf-headnote..

[14] The Florida Cancer Data System. https://fcds.med.miami.edu/inc/welcome.shtml.

[15] Mandal A. (2013). Factors Affecting African-American Health: Empowering the Community with Health Literacy. Bioprocess Biotech..

[16] Riley W. (2017). Health Disparities: Gaps in Access, Quality and Affordability of Medical Care. Trans. Am. Clin. Climatol. Assoc..

[17] Holick C.N., Giovannucci E.L., Rosner B., Stampfer M.J., Michaud D.S. (2007). Prospective Study of Cigarette Smoking and Adult Glioma: Dosage, Duration, and Latency. Neuro Oncol..

[18] Shao C., Zhao W., Qi Z., He J. (2016). Smoking and Glioma Risk: Evidence from a Meta-Analysis of 25 Observational Studies. Medicine.

[19] WWAMI Rural Health Research Center Rural Urban Commuting Areas (RUCA). http://http://depts.washington.edu/uwruca/ruca-data.php.

